# Exploring the potential effect of *Ocimum sanctum *in vincristine-induced neuropathic pain in rats

**DOI:** 10.1186/1749-7221-5-3

**Published:** 2010-01-25

**Authors:** Gurpreet Kaur, Amteshwar Singh Jaggi, Nirmal Singh

**Affiliations:** 1 Department of Pharmaceutical Sciences and Drug Research, Punjabi University, Patiala, India

## Abstract

The present study was designed to investigate the ameliorative potential of *Ocimum sanctum *and its saponin rich fraction in vincristine-induced peripheral neuropathic pain in rats. Peripheral neuropathy was induced in rats by administration of vincristine sulfate (50 μg/kg *i.p.*) for 10 consecutive days. The mechanical hyperalgesia, cold allodynia, paw heat hyperalgesia and cold tail hyperalgesia were assessed by performing the pinprick, acetone, hot plate and cold tail immersion tests, respectively. Biochemically, the tissue thio-barbituric acid reactive species (TBARS), super-oxide anion content (markers of oxidative stress) and total calcium levels were measured. Vincristine administration was associated with the development of mechanical hyperalgesia, cold allodynia, heat and cold hyperalgesia. Furthermore, vincristine administration was also associated with an increase in oxidative stress and calcium levels. However, administration of *Ocimum sanctum *(100 and 200 mg/kg *p.o.*) and its saponin rich fraction (100 and 200 mg/kg *p.o.*) for 14 days significantly attenuated vincristine-induced neuropathic pain along with decrease in oxidative stress and calcium levels. It may be concluded that *Ocimum sanctum *has ameliorative potential in attenuating chemotherapy induced-painful neuropathic state, which may be attributed to decrease in oxidative stress and calcium levels. Furthermore, saponin rich fraction of *Ocimum sanctum *may be responsible for its noted beneficial effect in neuropathic pain in rats.

## Introduction

Neuropathic pain has been described as "the most terrible of all tortures which a nerve wound may inflict" [1]. Despite progress in the understanding of this syndrome, the mechanistic details underlying the disease remain elusive. Neuropathic pain is generally characterized by the sensory abnormalities such as unpleasant abnormal sensation (dysesthesia), an increased response to painful stimuli (hyperalgesia), and pain in response to a stimulus that does not normally provoke pain (allodynia) [2]. Peripheral neuropathic pain is frequently observed in patients with cancer, AIDS, long standing diabetes, lumbar disc syndrome, herpes infection, traumatic spinal cord injury, multiple sclerosis and stroke [3]. Moreover, post-thoracotomy, post-herniorrhaphy, post-mastectomy and post-sternotomy are some other conditions often associated with peripheral neuropathy pain [4].

Chemotherapeutic drugs such as vincristine, paclitaxel, oxaliplatin, etc. are widely used in management of cancers especially Hodgkins lymphoma, non-Hodgkins lymphoma and leukemia. Unfortunately, these anti-cancer agents have been documented to produce dose and duration dependent neurotoxicity and painful neuropathy [5] which limit their full exploitation in the management of tumors. Vincristine is unique among the chemotherapeutic agents that it produces predictably and uniformly neurotoxicity in all the patients even at the therapeutic doses [6]. This peripheral neuropathy is dose-related with a marked variability in individual susceptibility. After stopping vincristine administration, partial or complete clinical recovery follows which takes several months.

Though some drugs have been found to be effective in managing the symptoms of neuropathy, yet their full clinical exploitation is limited due to wide spectrum of adverse effects associated with their clinical use. Moreover, none of the medications, assessed in randomized controlled studies conducted, has been found to be effective in injury induced and chemotherapy-induced neuropathic pain [7]. Therefore, there has been an urgent need of alternative medicine for managing neuropathy particularly in injury and chemotherapy-induced neuropathic pain, for which the best option may be to look back at the traditional medicines.

*Ocimum sanctum *(L.), (syn, Tulsi) is an indigenous plant commonly found in India and is recommended in the Ayurveda for the treatment of bronchial asthma, malaria, dysentery, skin diseases, arthritis, painful eye diseases, chronic fever and insect bite. Experimental reports have indicated its protective effects against genotoxicants, chemical carcinogens, ischaemic cerebral injury [8], ischaemia-reperfusion and isoproterenol-induced myocardial damage. Moreover, its anti-convulsant [9], hepato-protective, immuno-modulatory, anti-ulcer, anti-diabetic, anti-hypercholesterolaemic, chemo-protective, nootropic, antitussive, anti-inflammatory, wound healing, anti-tumorigenesis, anthelmintic, anti-bacterial, anti-giardial [10], anti-anxiety [11], and anti-stress activities have also been documented. Traditionally, *Ocimum sanctum *has been used as nerve tonic and to alleviate joint pain, headache and muscular pain particularly in South India. Recently, from our laboratory it has been documented that *Ocimum sanctum *has ameliorative potential in attenuating sciatic nerve transection-induced neuropathic pain [12].

Saponins are important phyto-constituents present in different plants including *Ocimum sanctum *in which these constitute an important chemical class and include pentacyclic triterpenoids saponins such as ursolic acid, oleanolic acid [13,14]. Number of studies have shown that saponins exert diverse biological actions such as anti-hypertensive [15], anti-cancer [16], anti-convulsant [17], anti-diabetic [18], anti-amnestic [19], hypocholesterolaemic [20] and neuroprotective [21]. Furthermore, saponins have also been shown to exhibit anti-nociceptive actions [22,23]; alleviate neuropathic pain in long standing diabetes [24] and nerve entrapment induced facial paralysis (Bell's palsy) [25].

The present study was designed to investigate the ameliorative role of *Ocimum sanctum *in vincristine-induced neuropathy and to further explore the contributory role of saponins in *Ocimum sanctum *mediated beneficial effect in neuropathic pain in rats.

## Materials and methods

### Experimental Animals

Wistar albino rats weighing 180-250 g, maintained on standard laboratory diet (Kisan Feeds Ltd., Mumbai, India) and having free access to tap water were employed in the present study. They were housed in the departmental animal house and were exposed to normal cycle of light and dark. The experimental protocol was approved by the Institutional Animal Ethics Committee (IAEC) and the care of the animals was carried out as per the guidelines of the Committee for the Purpose of Control and Supervision of Experiments on Animals (CPCSEA), Ministry of Environment and Forest, Government of India (Reg. No- 107/1999/CPCSEA).

### Drugs and Chemicals

Vincristine sulfate (Chandra Bhagat Pharma Pvt. Ltd., Mumbai), was dissolved in normal saline. All the reagents used in the present study were of analytical grade.

### Plant material

Fresh leaves of *Ocimum sanctum *were collected from Patiala and authenticated through Botany Department, Punjabi University, Patiala. The Plant sample has been kept in Voucher specimen (PUP-039/2008-2009) at Punjabi university, Patiala. The fresh leaves of *Ocimum sanctum *were shed dried at room temperature and reduced to coarse powder. The powder was extracted with mixture of methanol: water (3:1). The solvent was completely removed at 50°C under reduced pressure. The yield of the extract was 13% (w/w) in terms of dried starting material. The extract was standardized using HPTLC finger-printing taking chloroform and methanol (80:20) as mobile phase and using anisaldehyde sulphuric acid as spraying agent. The bands were detected at 254 nm, 366 nm and under white light (Table 1). The saponin rich fraction was extracted from the concentrated hydro-alcoholic extract as described earlier [26,27]. Briefly, the hydro-alcoholic extract was decanted by n-hexane followed by extraction with n-butanol. After three successive extractions with n-butanol, the resulting solutions were combined and the butanol was completely removed at 50°C under reduced pressure to collect the residue rich in saponin. The saponins were identified using froth test and triterpenoid saponins were identified using Lieberman-Burchard test.

**Table 1:** HPTLC fingerprinting of hydro-alcholic extract of *Ocimum sanctum *taking chloroform and methanol (80:20) as mobile phase and using anisaldehyde as spraying agent. The values indicate the R_f _values of the separated bands.

S. No of Resolving Bands	UV 254 nm	UV 366	Under white light
1.	-	-	0.44

2.	-	-	0.60

3.	-	-	0.71

4.	-	-	0.78

5.	-	-	0.85

### Induction of Neuropathic Pain by Vincristine

Peripheral neuropathy was induced in rats by administration of vincristine sulfate (50 μg/kg *i.p.*) for 10 consecutive days as described previously [28].

### Behavioral Examination

#### Paw Cold-Allodynia (Acetone Drop Test)

The cold allodynia was assessed by spraying a 100 μL of acetone onto the planter surface of the paw, without touching the skin. The duration of withdrawal response was recorded with an arbitrary minimum value of 0.5 s and a maximum value of 20 s [29].

#### Paw Heat-Hyperalgesia (Hot Plate Test)

The thermal nociceptive threshold, as an index of thermal hyperalgesia, was assessed by the Eddy's hot plate, which is an instrument designed by Eddy and co-workers to assess thermal sensitivity. The plate was pre-heated and maintained at a temperature of 52.5 ± 2.0°C. The rat was placed on the hot plate and nociceptive threshold, with respect to licking of the hind paw, was recorded in seconds. The cut-off time of 20 s was maintained [30].

#### Mechanical hyperalgesia: (Pin prick test)

The mechanical hyperalgesia was assessed by the pinprick test as described by Erichsen and Blackburn-Munro [31]. The surface of the injured hind paw was touched with the point of the bent gauge needle (at 90° to the syringe) at intensity sufficient to produce a reflex withdrawal response in normal non-operated animals, but at an intensity which was insufficient to penetrate the skin. The duration of the paw withdrawal was recorded in seconds with a stopwatch. A cut-off time of 20 s was maintained.

#### Tail Cold-Hyperalgesia Test (Tail Immersion Test)

The tail cold-hyperalgesia was noted by immersing a terminal part of the tail (1 cm) in the water, maintained at a temperature of 0-4°C. The tail withdrawal latency was recorded and a cut-off time of 20 s was maintained [32].

### Biochemical Estimation

All the groups of animals were sacrificed after 14^th ^day by cervical dislocation and the sciatic nerve was isolated immediately [30,33]. The uniformity among the different nerve samples was maintained by taking the constant weight of the respective samples. The excised sciatic nerve homogenate (10% w/v) was prepared with 0.1 M Tris- HCl buffer (pH 7.4). The tubes with homogenate were kept in ice water for 30 minutes and centrifuged at 4°C (2500 rpm, 10 min). The supernatant of homogenate was separated, and employed to estimate total protein content, TBARS, superoxide anion concenteration and total calcium content.

#### Estimation of the protein content

The protein concentration in the sciatic nerve was estimated according to the method of Lowry *et al.*, [34] using bovine serum albumin as a standard. The absorbance was determined spectrophotometrically at 750 nm.

#### Estimation of thio-barbituric acid reactive substances

The estimation of lipid peroxidation in the sciatic nerve was done by measuring the thio-barbituric acid reactive substances by the method of Okhawa *et al*. [35]. The absorbance was measured spectrophotometrically at 532 nm. The concentration was expressed in terms of nmol of thio-barbituric acid reactive substances/mg protein.

#### 2.6.3. Estimation of superoxide anion generation

The superoxide anion generation in the sciatic nerve was estimated in terms of measuring reduced nitroblue tetrazolium (NBT) [36]. The absorbance of formazan was determined spectrophotometerically at 540 nm.

#### Estimation of total calcium

The total calcium levels were estimated in the sciatic nerve as described earlier [33,37]. Briefly, the sciatic nerve homogenate was mixed with 1 ml of trichloroacetic acid (4%) in ice cold conditions and centrifuged at 1500 g, for 10 minutes. The clear supernatant was used for the estimation of total calcium ion by atomic emission spectroscopy at 556 nm.

### Experimental Protocol

Nine groups, each group comprising six Wistar albino rats, were employed in the present study.

#### Group I: Normal control group

Rats were not subjected to any treatment and were kept for 14 days. The behavioral tests were employed on different days *i.e.*, day 2^nd^, 6^th^, 8^th ^and 14^th^. All the animals were sacrificed at end of the 14^th ^day and the biochemical analysis was done for estimation of protein content, TBARS, superoxide anion and total calcium.

#### Group II: Vincristine control group

Vincristine (50 μg/kg, *i.p.*) was administered to normal rats for 10 consecutive days (1-10). The behavioral tests were assessed starting on days 2^nd^, 6^th^, 8^th ^and 14^th^. At the end of 14^th ^day, the animals were sacrificed and biochemical analysis was done as described in group I.

#### Group III: Vehicle in vincristine control group

Rats were administered carboxymethylcellulose suspension (0.5% w/v, *p.o.*) 2 h before each vincristine injection, for 14 consecutive days (1-14). The behavioral tests and biochemical parameters were assessed as mentioned in group I.

#### Group IV: Hydro-alcoholic extract of *Ocimum sanctum per se*

The hydro-alcoholic extract of *Ocimum sanctum *(200 mg/kg *p.o.*) was administered to normal rats for 14 consecutive days, starting from the day one. The behavioral tests and the biochemical parameters were assessed as described in group I.

#### Group V: Saponin rich extract of *Ocimum sanctum per se*

The saponin rich extract of *Ocimum sanctum *(200 mg/kg *p.o.*) was administered to normal rats for 14 consecutive days, starting from the day one. The behavioral tests and the biochemical parameters were assessed as described in group I.

#### Group VI and VII: Hydro-alcoholic extract of *Ocimum sanctum *(100; 200 mg/kg p.o.) in vincristine control group

The hydro-alcoholic extract of *Ocimum sanctum *(100; 200 mg/kg *p.o.*) was administered for 14 consecutive days, starting from the day one, 2 h prior to vincristine administration. The behavioral tests and the biochemical parameters were assessed as described in group I.

#### Group VIII and IX: Saponin rich fraction of *Ocimum sanctum *(100; 200 mg/kg p.o.) in vincristine control group

The saponin rich fraction of *Ocimum sanctum *(100 and 200 mg/kg *p.o.*) was administered for 14 consecutive days, starting from the day one, 2 h prior to vincristine administration. The behavioral tests and the biochemical parameters were assessed as described in group I.

### Statistical Analysis

All the results were expressed as mean ± standard error mean (S.E.M.). The data of behavioral results was statistically analyzed by two-way ANOVA followed by Bonferonni's post test by using Graph pad prism Version-5.0 software. The data of biochemical results was statistically analyzed by one-way ANOVA followed by Tukey's multiple range test by using Sigmastat Version-2.0 software. The *p-value *< 0.05 was considered to be statistically significant.

## Results

### Effect of *Ocimum sanctum *and its Saponin Rich Fraction on Cold Allodynia in Neuropathic Pain

**Figure 1:**
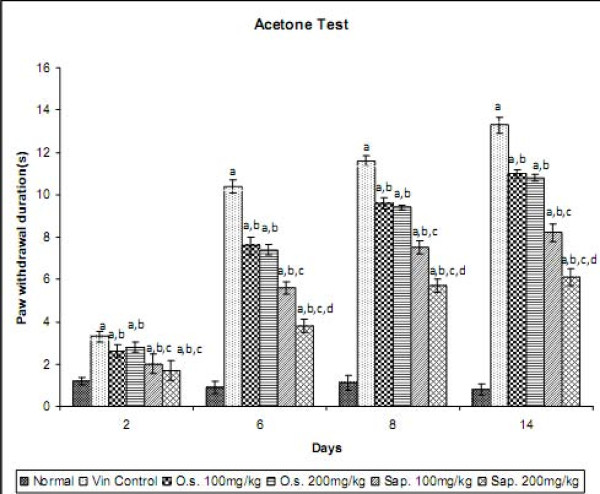
**Effect of *Ocimum sanctum *and its saponin rich fraction on cold allodynia in vincristine-induced neuropathic pain**. Vin.: vincristine; O.s.: *Ocimum sanctum*; Sap.: saponin rich extract of *Ocimum sanctum*. Data were expressed as mean ± S.E.M., n = 6 rats per group. a = p < 0.05 Vs normal control group, b = p < 0.05 Vs vincristine control group, c = p < 0.05 Vs *Ocimum sanctum *100 and 200 mg/kg group, d = p < 0.05 Vs saponin rich fraction 100 mg/kg group.

Vincristine administration resulted in the development of cold allodynia as reflected by an increase in the duration of hind paw withdrawal, when compared to normal control group. However, pre-treatment with *Ocimum sanctum *(100 and 200 mg/kg *p.o.*) and its saponin rich fraction (100 and 200 mg/kg *p.o.*) significantly attenuated vincristine-induced increase in the withdrawal duration of the hind paw in response to non-noxious cold stimuli. The effect of saponin rich fraction in attenuating cold allodynia was significantly higher than the hydro-alcoholic extract at the same dose levels (Figure 1). Vehicle administration did not modulate the behaviour in response to non-noxious cold stimulus in animals subjected to peripheral neuropathy. *Per se *administration of *Ocimum sanctum *and its saponin rich fraction also did not produce alterations in the normal rats.

### Effect of *Ocimum *sanctum and its saponin rich fraction on mechanical hyperalgesia in Neuropathic Pain

**Figure 2:**
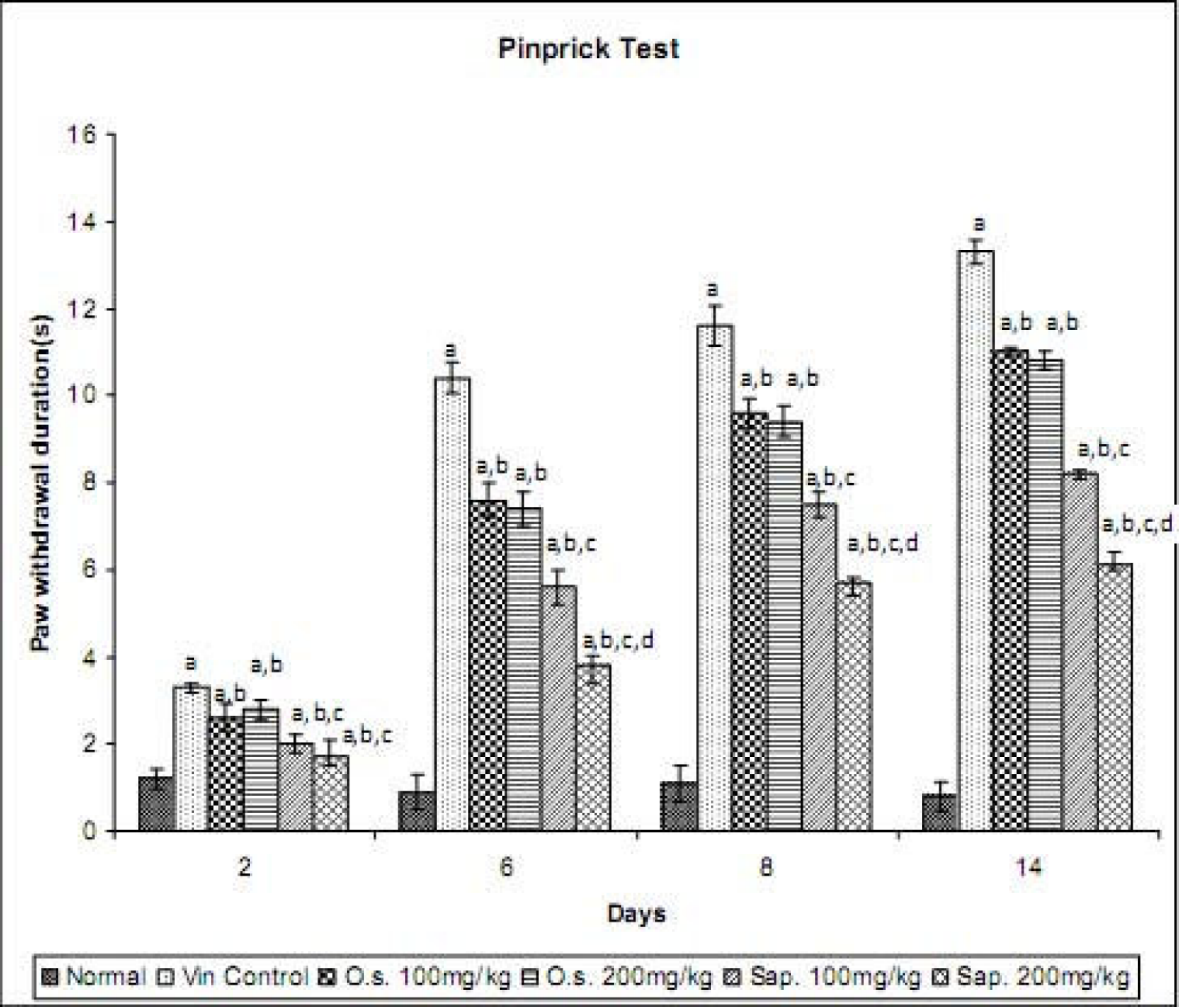
**Effect of *Ocimum sanctum *and its saponin rich fraction on mechanical hyperalgesia in vincristine-induced neuropathic pain**. Vin.: vincristine, O.s.: *Ocimum sanctum*, Sap.: saponin rich extract of *Ocimum sanctum*. Data were expressed as mean ± S.E.M., n = 6 rats per group. a = p < 0.05 Vs normal control group, b = p < 0.05 Vs vincristine control group, c = p < 0.05 Vs *Ocimum sanctum *100 and 200 mg/kg group, d = p < 0.05 Vs saponin rich fraction 100 mg/kg group.

Vincristine administration was associated with the development of mechanical hyperalgesia as reflected by an increase in the hind paw withdrawal duration, when compared to normal control group. Treatment with *Ocimum sanctum *(100 and 200 mg/kg *p.o.*) and its saponin rich fraction (100 and 200 mg/kg *p.o.*) significantly attenuated vincristine-induced increase in withdrawal duration of the hind paw in response to noxious mechanical stimuli. The effect of saponin rich fraction in attenuating mechanical hyperalgesia was significantly higher than the hydro-alcoholic extract at the same dose levels (Figure 2). Vehicle administration did not modulate behaviour in response to noxious mechanical stimulus in animals subjected to peripheral neuropathy. *Per se *administration of *Ocimum sanctum *and its saponin rich fraction also did not produce alterations in the normal rats.

### Effect of *Ocimum *sanctum and its saponin rich fraction on paw heat and cold tail hyperalgesia in Neuropathic Pain

**Figure 3:**
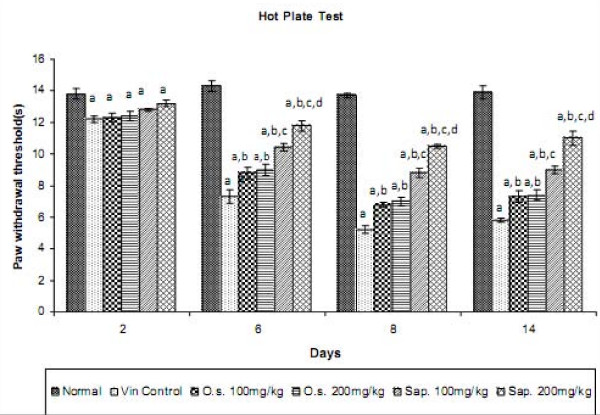
**Effect of *Ocimum sanctum *and its saponin rich fraction on paw heat hyperalgesia in vincristine-induced neuropathic pain**. Vin.: vincristine, O.s.: *Ocimum sanctum*, Sap.: saponin rich extract of *Ocimum sanctum*. Data were expressed as mean ± S.E.M., n = 6 rats per group. a = p < 0.05 Vs normal control group, b = p < 0.05 Vs vincristine control group, c = p < 0.05 Vs *Ocimum sanctum *100 and 200 mg/kg group, d = p < 0.05 Vs saponin rich fraction 100 mg/kg group.

**Figure 4:**
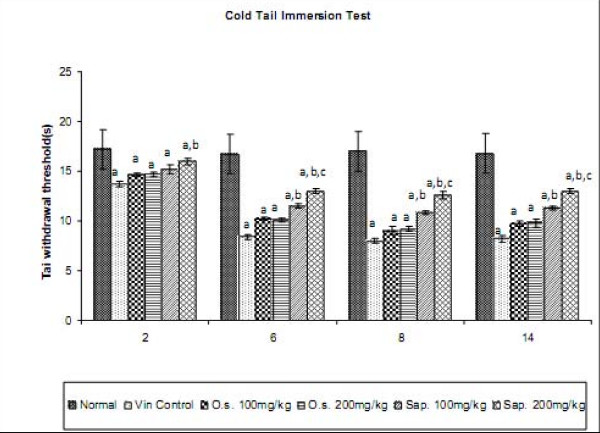
**Effect of *Ocimum sanctum *and its saponin rich fraction on tail cold hyperalgesia in vincristine-induced neuropathic pain**. Vin.: vincristine, O.s.: *Ocimum sanctum*, Sap.: saponin rich extract of *Ocimum sanctum*. Data were expressed as mean ± S.E.M., n = 6 rats per group. a = p < 0.05 Vs normal control group, b = p < 0.05 Vs vincristine control group, c = p < 0.05 Vs *Ocimum sanctum *100 and 200 mg/kg group.

Vincristine administration led to the development of paw heat and tail cold hyperalgesia as reflected by decrease in the withdrawal threshold of the hind paw and tail respectively, when compared to normal control group. However, treatment with *Ocimum sanctum *(100 and 200 mg/kg *p.o.*) and its saponin rich fraction (100 and 200 mg/kg *p.o.*) significantly attenuated vincristine-induced decrease in the withdrawal latency in response to noxious thermal stimuli. The effect of saponin rich fraction in attenuating thermal hyperalgesia was significantly higher than the hydro-alcoholic extract at the same dose levels (Figures 3 and 4). Vehicle administration did not modulate behaviour in response to noxious thermal stimulus in animals subjected to peripheral neuropathy. *Per se *administration of *Ocimum sanctum *and its saponin rich fraction also did not produce alterations in the normal rats.

### Effect of *Ocimum *sanctum and its saponin rich fraction on oxidative stress markers and total calcium content in Neuropathic Pain

Vincristine administration resulted in an increase in the oxidative stress markers and total calcium content as reflected by an increase in the tissue thio-barbituric acid reactive substances, superoxide anion content and total calcium levels, when compared to normal control group. However, treatment with *Ocimum sanctum *(100 and 200 mg/kg *p.o.*) and its saponin rich fraction (100 and 200 mg/kg *p.o.*) significantly attenuated the vincristine-induced increase in oxidative stress markers and total calcium levels. The effect of saponin rich fraction in attenuating the rise in the levels of oxidative stress markers and the calcium levels was significantly higher than the hydro-alcoholic extract at the same dose levels. Vehicle administration did not modulate alterations in the levels of oxidative stress markers and total calcium content in animals subjected to peripheral neuropathy. *Per se *administration of *Ocimum sanctum *and its saponin rich fraction also did not produce any biochemical alterations in the normal rats (Table 2).

**Table 2:** Effect of *Ocimum sanctum *and its saponin rich fraction on thio-barbituric acid reactive substances, superoxide anion content and total calcium in vincristine-induced neuropathic pain.

Groups	Doses	Total Protein (mg/g of tissue)	TBARS (nmol/mg of protein)	Superoxide Anion Content (pmol/min/mg of protein)	Total Calcium (ppm/mg of protein)
Normal Control	-	5.17 ± 0.23	5.97 ± 0.26	0.15 ± 0.02	4.97 ± 0.21

Vincristine Control	-	5.36 ± 0.13	8.28 ± 0.24^a^	0.79 ± 0.05^a^	32.20 ± 0.18^a^

Vehicle in Vincristine Control	-	5.29 ± 0.17	8.24 ± 0.22^a^	0.81 ± 0.03^a^	32.02 ± 0.16 ^a^

*Ocimum sanctum *per se	200 mg/kg	5.21 ± 0.15	6.00 ± 0.28	0.16 ± 0.01	5.09 ± 0.15

Saponin rich fraction per se	200 mg/kg	5.30 ± 0.14	6.06 ± 0.24	0.14 ± 0.02	4.92 ± 0.17

*Ocimum sanctum *in vincristine control	100 mg/kg	5.33 ± 0.22	7.56 ± 0.16^a, b^	0.56 ± 0.02^a, b^	29.5 ± 0.18^a, b^

*Ocimum sanctum *in vincristine control	200 mg/kg	5.26 ± 0.16	7.52 ± 0.15^a, b^	0.54 ± 0.03^a, b^	29.8 ± 0.20^a, b^

Saponin rich fraction in vincristine control	100 mg/kg	5.35 ± 0.13	6.90 ± 0.19^a, b, c^	0.38 ± 0.04^a, b, c^	21.1 ± 0.19^a, b, c^

Saponin rich fraction in vincristine control	200 mg/kg	5.29 ± 0.14	6.28 ± 0.21^a, b, c, d^	0.25 ± 0.02^a, b, c, d^	12.2 ± 0.13^a, b, c, d^

Data were expressed as mean ± S.E.M., n = 6 rats per group a = p < 0.05 Vs normal control group, b = p < 0.05 Vs vincristine control group, c = p < 0.05 Vs *Ocimum sanctum*100 and 200 mg/kg group, d = p < 0.05 Vs saponin rich fraction 100 mg/kg group.

## Discussion

In the present investigation, vincristine (50 μg/kg, *i.p.*) administration for 10 days led to significant development of cold allodynia, mechanical, tail cold and paw heat hyperalgesia. The observed behavioral alterations in this study are in consistent with the earlier reports documenting the development of pain symptoms with vincristine administration [28,33]. Vincristine has been widely employed for the management of number of cancers including Hodgkin's disease. However, its clinical application has been limited due to unavoidable painful sensorimotor neuropathy, observed in about half of the patients on vincristine treatment. Clinically, the neuropathy is characterized by paresthesias in hand and feet and the pinprick and thermal senses are more affected than vibration senses. Binding of vincristine to β-tubulin with subsequent disruption of microtubules has been documented for its anti-tumor actions and the same is also assumed to produce neuro-toxicity by axonal degeneration.

However, pretreatment with *Ocimum sanctum *significantly attenuated vincristine-induced alterations in pain perception in response to noxious as well as non-noxious stimuli, suggesting that *Ocimum sanctum *may be employed to limit the painful symptoms associated with chemotherapy treatment. Traditionally, *Ocimum sanctum *has been used as a nerve tonic to alleviate disorders related to nerves. Recently, it has been reported from our laboratory that *Ocimum sanctum *attenuates the neuropathic pain in sciatic nerve transection model [12].

*Ocimum sanctum *is a rich source of a number of saponins and the most important of them are ursolic acid and oleanolic acid. These saponins have been reported to possess beneficial effects in number of disorders such as in amnesia [38], hypertension [39], myocardial ischemia [40], and cancer [41]. Furthermore, the saponins have also shown the beneficial effects in relieving nociceptive pain [22,23] as well as neuropathic pain in diabetes [24] and facial paralysis (Bell's palsy) due to nerve entrapment [25]. Therefore, to explore the chemical class of *Ocimum sanctum *responsible of its beneficial effect its saponin rich fraction was evaluated in neuropathic pain. In the present investigation, pre-treatment with the saponin rich fraction of *Ocimum sanctum *attenuated vincristine-induced behavioral alterations in neuropathic pain. Therefore, it may be proposed that saponins are the principal components responsible for the noted beneficial effects of *Ocimum sanctum *in neuropathic pain.

Furthermore, in this study vincristine administration was associated with the rise in the oxidative stress (rise in thio-barbituric acid reactive substances and superoxide anion content) and total calcium content. It has also been documented that oxidative stress and an increase in calcium levels play a critical role in chemotherapy associated neuropathic pain including vincristine [28,33,42]. However, treatment with *Ocimum sanctum *and its saponin rich fraction attenuated vincristine associated increase in oxidative stress and calcium levels. *Ocimum sanctum *has a well documented antioxidant effect [10,43] and also decreases the calcium levels [12].

Free radicals have been well documented to increase calcium levels [44,45]. Therefore, the observed decrease in calcium levels with *Ocimum sanctum *may possibly be attributed to its antioxidant effects. However, it may also be possible that direct action of *Ocimum sanctum *is responsible for the observed decrease in the calcium levels. Therefore, the observed decrease in calcium levels due to *Ocimum sanctum *may be either due to direct action or secondary to decrease in oxidative stress. However, data of the present study is still insufficient to elaborate the precise mechanism of *Ocimum sanctum *mediated decrease in calcium levels. Furthermore, saponins have also been reported to decrease oxidative stress [46] and calcium levels [47-49]. Based on these, it may be proposed that *Ocimum sanctum *has potential in ameliorating the painful symptoms in vincristine-induced peripheral neuropathy and saponins may be the principal chemical class responsible for its beneficial effect in neuropathic pain. Furthermore, the pain relieving effects of *Ocimum sanctum *and its saponin rich fraction may be due to attenuation of nerve injury inciting agent-induced increased levels of calcium and free radicals.

## Competing interests

The authors declare that they have no competing interests.

## Authors' contributions

GK carried out experimental studies including induction of neuropathy, behavioral and biochemical testing. ASJ carried out data analysis including the statistical analysis and participated in critical intellectual discussion and designing of the experiments. NS conceived the idea, coordinated the study, carried our data interpretation and drafted the manuscript. All authors read and approved the final manuscript.
